# A novel MYD88 mutation, L265RPP, in Waldenström macroglobulinemia activates the NF-κB pathway to upregulate Bcl-xL expression and enhances cell survival

**DOI:** 10.1038/bcj.2015.36

**Published:** 2015-05-15

**Authors:** T Nagao, G Oshikawa, S Ishida, H Akiyama, Y Umezawa, A Nogami, T Kurosu, O Miura

**Affiliations:** 1 Department of Hematology, Graduate School of Medical and Dental Sciences, Tokyo Medical and Dental University, Tokyo, Japan

Waldenström macroglobulinemia (WM) is a distinct clinicopathological entity defined by a low-grade B-cell lymphoma, lymphoplasmacytic lymphoma, infiltrating the bone marrow and producing a monoclonal immunoglobulin of the IgM class.^1^ Recently, a distinct mutation in MYD88, L265P, has been found in more than 90% of patients with WM.^2^ On the other hand, various MYD88 mutations have been found in about 40% of cases with activated B-cell-like diffuse large B-cell lymphoma (ABC-DLBCL), with MYD88 L265P constituting 75–80% of these mutations.^3^ However, MYD88 L265P represents almost the sole MYD88 mutation reported so far in WM and in 50–60% of patients with the IgM-type monoclonal gammopathy of undetermined significance, which may precede WM.^1, 2, 4^ This mutation has been found in <10% of patients with other low-grade B-cell neoplasms showing plasmacytic differentiation and producing a monoclonal IgM protein, such as splenic marginal zone lymphoma, chronic lymphocytic leukemia and IgM-secreting multiple myeloma. Thus, detection of this mutation is valuable for the differential diagnosis of WM from these low-grade B-cell neoplasms, leading to the development of highly sensitive allele-specific PCR (AS-PCR) methods to detect specifically the MYD88 L265P mutation.^1, 5, 6, 7, 8^

MYD88 is an adaptor protein that mediates Toll-like receptors (TLRs) and interleukin-1 receptor (IL-1R) signaling regulating diverse immune responses.^9, 10^ MYD88, as well as TLRs and IL-1R, possesses a Toll–IL-1 receptor (TIR) domain and is recruited to these receptors upon stimulation of their ligands through homophilic interaction between the respective TIR domains. MYD88 then interacts with and activates the IRAK1 and IRAK4 serine/threonine kinases, ultimately leading to NF-κB activation via IκBα phosphorylation. The L265P mutation in the TIR domain has been postulated to change the structure of MYD88 to induce constitutive formation of the MYD88/IRAK complex to activate the NF-κB pathway to stimulate proliferation and to suppress apoptosis of B cells.^1, 2, 3^ MYD88 L265P has also been reported to promote NF-κB activation by binding and activating BTK and to activate the Jak-STAT3 pathway as additional downstream effects.^3, 11^ Through these mechanisms, MYD88 mutations have been implicated in the pathogenesis and development of therapy resistance in B-cell neoplasms, mainly WM and ABC-DLBCL. Thus, MYD88 mutants, as well as their downstream effectors, may represent promising therapeutic targets for these diseases and need to be further investigated in detail.

We have developed a very simple AS-PCR method that could detect the MYD88 L265P mutation when present in >1% of cells and examined the bone marrow cells from five patients with WM (Supplementary Materials). We failed to detect this mutation in one out of the five patients examined (Supplementary Figure S1), although this patient showed typical clinical features of WM (Supplementary Materials) and abnormal plasmacytic cells constituted >5% of the bone marrow cells. Thus, we directly sequenced the PCR products coding for *MYD88* exon 5 derived from genomic DNA, as well as cDNA from the bone marrow cells of the patient. Because a region around the sequences coding for MYD88 L265 gave mixed and ambiguous signals (data not shown), we next sequenced and analyzed the subcloned full-length *MYD88* RT-PCR products, as well as genomic DNA PCR products, and found a novel mutation of *MYD88*, c.792_794delACTinsGCGGCCCCC (Figure 1a), causing the substitution of L265 with RPP (L265RPP) (Figure 1a).

Because the MYD88 L265RPP mutation has never been reported in WM or in any other B-cell malignancies, we analyzed the effects of this novel mutation comparably with MYD88 L265P by constructing retroviral expression plasmids for both of these mutants, as well as for wild-type MYD88, tagged N-terminally with the FLAG epitope in the pMIG vector. We infected the BJAB cells, a model lymphoma cell line with low endogenous NF-κB activity used in studies for MYD88 mutations,^3^ with these retroviral vectors and selected infected cells by sorting cells expressing GFP. As shown in Figure 1b, western blot analysis revealed that MYD88 L265RPP and, to a lesser extent, MYD88 L265P were expressed at lower levels as compared with wild-type MYD88 in BJAB cells, which expressed comparable levels of GFP (data not shown), thus suggesting that these mutants may be unstable in these cells. To address this possibility, we treated these BJAB cells with cycloheximide to shut down translation and examined its effect on the expression levels of these MYD88 proteins. As shown in Figure 1c, expression levels of MYD88 L265RPP and, to a lesser extent, MYD88 L265P were more rapidly reduced by cycloheximide as compared with that of wild-type MYD88, thus indicating that these mutants are more rapidly degraded in cells as compared with wild-type MYD88. To explore the mechanisms involved in degradation of these mutant MYD88 proteins, we next examined the effects of the proteasome inhibitor bortezomib, which is used also for treatment of WM,^12^ on expression of the MYD88 proteins. As shown in Figure 1d, the expression level of MYD88 L265RPP was more significantly increased by bortezomib as compared with that of wild-type MYD88, whereas that of MYD88 L265P did not show any significant change. These results suggest that MYD88 L265RPP may be rapidly degraded through the proteasome pathway sensitive to inhibition by bortezomib in a different manner as compared with MYD88 L265P.

Next, we examined the possible activation of the MYD88-IRAK1/4 and NF-κB pathway in BJAB cells expressing MYD88 L265RPP, as well as MYD88 L265P. First, we failed to detect the physical association of MYD88 L265RPP or MYD88 L265P with IRAK1 by coimmunoprecipitation experiments (negative data not shown). However, when stabilized by treatment with bortezomib, MYD88 L265RPP, but not MYD88 L265P or wild-type MYD88, was shown to associate prominently with slowly migrating IRAK1 species, which should represent IRAK1 hyper-phosphorylated by IRAK4 (Figure 2a).^3^ Moreover, in BJAB cells expressing MYD88 L265RPP, IκBα was phosphorylated to a much higher extent than in cells expressing wild-type MYD88 or MYD88 L265P, whereas its expression level was slightly decreased in cells expressing MYD88 L265RPP, as expected from its enhanced degradation after phosphorylation (Figure 2b). In agreement with this, NF-κB p65 was phosphorylated much more significantly in MYD88 L265RPP-expressing BJAB cells than in MYD88 L265P-expressing cells, in which phosphorylation of p65 was also conspicuously observed in accordance with a previous report (Figure 2c).^2^ Although these cells proliferated similarly in medium containing 10% fetal calf serum (FCS, negative data not shown), the XTT assay showed that viability of BJAB cells expressing MYD88 L265RPP, as well as MYD88 L265P, was less compromised by deprivation of FCS as compared with those overexpressing wild-type MYD88 or vector-control cells (Figure 2d). In accordance with this, BJAB cells expressing MYD88 L265RPP, as well as MYD88 L265P, underwent apoptosis less significantly than the control cells under these conditions (Figure 2e). Furthermore, bortezomib induced apoptosis less significantly in BJAB cells expressing MYD88 L265RPP or MYD88 L265P as compared with wild-type MYD88 (Figure 2f and g). To gain an insight into the molecular mechanisms underlying the prosurvival effect of MYD88 L265RPP, we examined its effect on the expression of Bcl-xL, which is an anti-apoptotic Bcl-2 family member previously implicated in the enhancement of survival by TLR signaling pathway involving MYD88 and NF-κB in CD4-positive T cells.^13^ As shown in Figure 2b, expression levels of Bcl-xL were increased in both MYD88 L265RPP- and L265P-expressing BJAB cells as compared with cells transduced with wild-type MYD88. Together, these data indicate that MYD88 L265RPP activates the MYD88-IRAK1/4 and NF-κB pathway more prominently than MYD88 L265P and shows comparable anti-apoptotic effects most likely through upregulation of Bcl-xL.

Because MYD88 L265P has been reported as the sole *MYD88* mutation in WM with the very high occurrence rates up to 100%, sensitive AS-PCR methods to detect this mutation, similar to the one we used in this study, have been developed to assist differential diagnosis of WM from other indolent lymphomas producing monoclonal IgM.^1, 5, 6, 7, 8^ However, the present study suggests that application of these methods alone fails to detect novel *MYD88* mutations that may exist in WM and might even misdiagnose WM patients lacking MYD88 L265P. Previously, the MYD88 S291C mutation, a recurrent mutation in DLBCL, has been reported in a patient with WM.^4^ Nevertheless, because this mutation was detected as a sub-clonal event in the presence of L265P, its significance has remained to be known. On the other hand, L265RPP is a novel mutation found as the sole MYD88 mutation in our case, and its potency to aberrantly activate MYD88 functions has been demonstrated in this study, thus suggesting that it is a driver mutation of tumor progression in WM.

Although MYD88 mutations other than L265P have been reported in various other B-cell malignancies, MYD88 L265P has been characterized as biologically most potent and unique in its ability to coordinate a stable signaling complex containing phosphorylated IRAK1.^3^ Our studies in BJAB cells suggest that MYD88 L265RPP may associate with IRAK1 more stably and phosphorylate IκBα more potently than MYD88 L265P to stimulate phosphorylation of IκBα and NF-κB p65, most likely through IKKα/β.^10, 14^ This may be owing to the more drastic alteration of structures surrounding L265 expected from the insertion of three irrelevant amino acids in L265RPP as compared with the single amino-acid substitution in L265P, which may also explain the difference in degradation through the ubiquitin-proteasome pathway. Thus, this mutant may provide an invaluable model system to investigate the molecular mechanisms underlying MYD88-mediated lymphoma pathogenesis, as well as therapy resistance, and to develop new therapeutic strategies against lymphoid malignancies with MYD88 mutations.

## Figures and Tables

**Figure 1:**
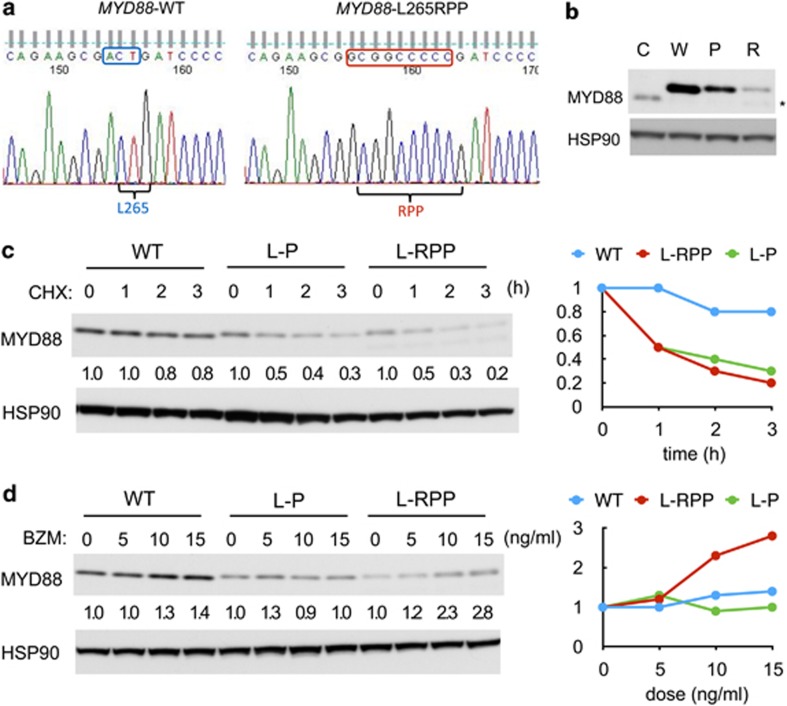
Analyses of DNA sequences of a novel MYD88 mutant, MYD88 L265RPP, and its stability, as well as degradation mechanism in BJAB cells. (**a**) Sequence analysis of *MYD88* transcripts obtained by RT-PCR from BJAB cells or the patient's bone marrow mononuclear cells. Nucleotide sequencing results of a subclone of RT-PCR products coding for wild-type MYD88 (MYD88-WT) from BJAB cells and that coding for MYD88 L265RPP from the clinical sample are shown, as indicated. The mutated nucleotide sequences (c.792_794delACTinsGCGGCCCCC) and their deduced amino-acid sequences are indicated by a box and letters in red, respectively, whereas the normal sequences corresponding to the mutation are indicated by those in blue. (**b**) BJAB cells infected with an empty vector (C) or with retroviral vectors expressing wild-type MYD88 (W), MYD88 L265P (P) and MYD88 L265RPP (R), as indicated, and sorted for GFP-expression were lysed and subjected to western blot analysis using antibodies against indicated proteins. HSP90 was used for loading control. An asterisk indicates the position of endogenous MYD88. (**c** and **d**) BJAB cells expressing wild-type MYD88 (WT), MYD88 L265P (L-P) or MYD88 L265RPP (L-RPP) were treated with 10 μg/ml cycloheximide (CHX) for indicated times (**c**) or with indicated concentrations of bortezomib (BZM) for 5 h (**d**) and lysed. Cell lysates were subjected to western blot analysis with antibodies against indicated proteins. Relative expression levels of MYD88 as compared with that in cells not treated with CHX or BZM were determined by densitometric analyses and indicated below each lane and graphically demonstrated.

**Figure 2:**
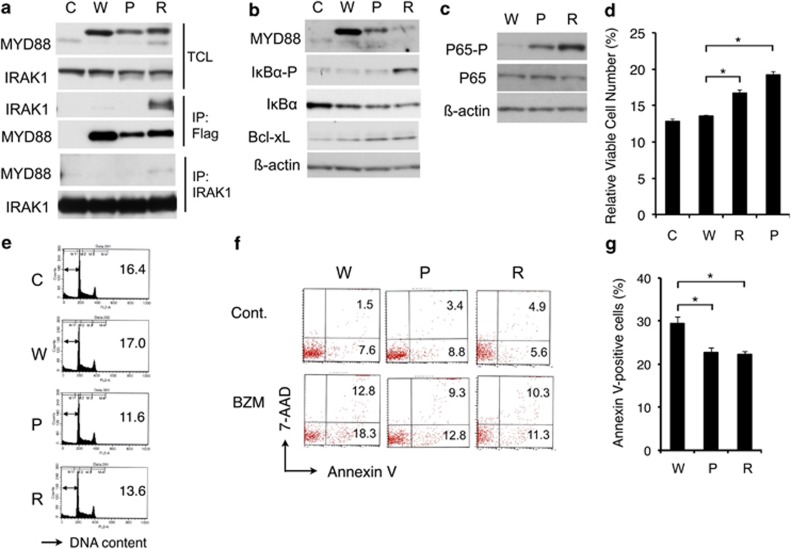
MYD88 L265RPP physically associates with IRAK1 and activates the NF-kB pathway to upregulate Bcl-xL expression and inhibits apoptosis in BJAB cells. (**a**) BJAB cells transduced with the control empty vector (C) or expression vectors for wild-type MYD88 (W), MYD88 L265P (P) or MYD88 L265RPP (R) were treated with 5 ng/ml bortezomib for 4 h and lysed. Cell lysates were subjected to immunoprecipitation with anti-Flag (Flag) or anti-IRAK1 (IRAK1), as indicted, and analyzed along with total cell lysates (TCL) by western blot analysis using antibodies against indicated proteins. (**b** and **c**) BJAB cells as described in **a** were lysed and subjected to western blot analysis using antibodies against indicated proteins. (**d**) BJAB cells as described in **a** were cultured with or without 10% FCS for 2 days, and viable cell numbers were measured by XTT colorimetric assay. The values demonstrated are the means±s.e. of triplicate determinations of cells cultured without FCS and are expressed as percentages of cell numbers cultured with 10% FCS. Asterisks indicate *P*<0.01, as determined by Student's *t*-test. (**e**) BJAB cells as described in **a** were cultured without 10% FCS for 2 days and analyzed for the cellular DNA content by flow cytometry. Percentages of apoptotic cells with sub-G1 DNA content are indicated. (**f** and **g**) BJAB cells as described in **a** were cultured with or without 5 ng/ml bortezomib (BZM) for 2 days and stained with anti-Annexin V-PE and 7-AAD to be analyzed by flow cytometry. Percentages of cells positive for both Annexin V and 7-AAD or Annexin V alone are indicated in **f**. Percentages of cells positive for Annexin V are plotted in **g**. The values demonstrated are the means±s.e. of triplicate determinations with asterisks indicating *P*<0.01.
