# Supplementary material for: A novel MYD88 mutation, L265RPP, in Waldenström macroglobulinemia activates the NF-κB pathway to upregulate Bcl-xL expression and enhances cell survival
Source: Blood Cancer J. 2015 May 15;5(5):e314–. doi: 10.1038/bcj.2015.36 (PMC4476015; doi:10.1038/bcj.2015.36)
Supplement: Supplementary Information [file bcj201536x1.doc]

**SUPPLEMENTARY INFORMATION**

**MATERIALS AND METHODS**

**Ethics Statement**

The study was approved by the ethical committee of Tokyo Medical and Dental University. Written informed consent was obtained from the patient in compliance with the Declaration of Helsinki.

**Case report**

A 58-year-old Japanese man was referred to our hospital because of general fatigue and inguinal lymph node swelling in September 2011. Laboratory tests revealed a mild anemia with hemoglobin 11.6 g/dL and an increase in the serum concentration of IgM to 1236 mg/dL (normal 28-177 mg/dL) with IgM-κ type M-protein revealed by immunoelectrophoresis using specific antibodies. The lymph node and bone marrow biopsies revealed infiltration of lymphoplasmacytic cells positive for CD19, CD20, and the immunoglobulin κ-light chain but negative for CD5 and CD23, which was compatible with pathological diagnosis of lymphoplasmacytic lymphoma. The cytogenetic analysis did not show any karyotypic abnormality. The patient was diagnosed with WM and treated with 6 courses of Rituximab-CHOP therapy on progression of general fatigue and anemia. He has gained a partial response and has not shown any progression of the residual disease.

**Cells and Reagents**

BJAB cells were obtained from the American Type Culture Collection. TMD8,[^1^](#_ENREF_1) an ABC-DLBCL cell line harboring the MYD88 L265P mutation,[^2^](#_ENREF_2) was kindly provided by Dr. Shuji Tohda. These cells were cultured in RPMI medium containing 10% FCS.

Cycloheximide was purchased from Sigma (St. Louis, MO, USA). Bortezomib was from Millennium Pharmaceuticals (Cambridge, MA, USA). Antibodies against MYD88, IRAK1, IκBα, and phospho-S32-IκBα, NF-κB p65, phospho-S536-NF-κB p65 were purchased from Cell Signaling (Beverly, MA, USA). Antibodies against ß-actin and FLAG were from Sigma. Antibodies against Bcl-xL and HSP90 were from BD Biosciences (San Jose, CA, USA) and Santa Cruz Biotechnology (Santa Cruz, CA, USA), respectively.

**Allele specific PCR**

We invented an allele specific genomic DNA PCR method to detect easily the *MYD88* L265P mutation. PCR from genomic DNA was performed by using mixture of a 5’ forward primer (MYD88-F1: 5‘-GGGATATGCTGAACTAAGTTGCCAC-3’) corresponding to sequences in intron 4 of *MYD88* and a 5’ forward primer (MYD88-F2: 5’-TTGCAGGTGCCCATCAGAAGCGTCC-3’) corresponding to the mutated sequence in exon 5 of *MYD88* along with a 3’ reverse primer (MYD88-R: 5’-GACGTGTCTGTGAAGTTGGCATCTC-3’) corresponding to sequences in intron 5. Using mixture of genomic DNA from MYD88-L265P-positive TMD8 cells and MYD88-L265P-negative BJAB cells, this method could detect the mutation present in 1 to 2 % of cells.

**Sequencing analyses of MYD88 and construction of expression plasmids**

For direct sequencing analysis of region encompassing *MYD88* exon 5, PCR products obtained from genomic DNA by using primers MYD88-F1 and MYD88-R were sequenced by using a primer, MYD88-F-Seq (5’-GCTGTTGTTAACCCTGGGGTTGAAG-3’). For direct sequencing analysis of MYD88 cDNA, RT-PCR products obtained by using a 5’ forward primer (MYD88-CF1: 5‘-AAGCGCTGGCAGACAATGCGACCCGAC-3’) and a 3’ reverse primer (MYD88-CR1: 5’-AGAACAGTCTTCAGGGCAGGGACAAG-3’) were similarly sequenced.

For sequencing analysis of cloned *MYD88* PCR products, genomic DNA fragments obtained by using MYD88-F1 and MYD88-R or full-length MYD88 cDNA fragments obtained by using MDY88-CF1 and MYD88-CR1 were subcloned into pGEM-T-Easy, and all the coding sequences were analyzed by using the SP6 and T7 primers and custom-made sequencing primers.

**Construction of expression plasmids and transduction into BJAB cells**

For construction of expression plasmids for wild-type MYD88, MYD88 L265P, and MYD88 L265RPP, full-length MYD88 cDNA fragments obtained from BJAB, TMD8, and the patient’s bone marrow cells, respectively, by RT-PCR using MYD88-CF1 and MYD88-CR1 were subcloned into pGEM-T-Easy and verified by sequencing, as described above. These cDNAs were N-terminally FLAG-tagged by the PCR-based method and subcloned into a retroviral bicistronic GFP-expression vector, pMIG (Addgene plasmid 9044), and all the coding sequences were verified by sequencing.

These retroviral expression plasmids were transduced into BJAB cells, and GFP-expressing cells were sorted by flow cytometry, as described previously.[^3^](#_ENREF_3)

**Analyses of Cell Proliferation, Viability, and Apoptosis**

Cell proliferation and viability were assessed by counting viable and nonviable cell numbers by the trypan blue dye exclusion method. Cell viability was calculated by dividing number of viable cells by that of total cells. Viable cell numbers were also assessed by the sodium 3’-[1-(phenylaminocarbonyl)-3,4-tetrazolium]-bis (4-methoxy-6-nitro)benzene sulfonic acid hydrate (XTT) colorimetric assay using the Cell Proliferation Kit II (Roche Molecular Biochemicals, Mannheim, Germany), according to the manufacture’s instructions.

For analysis of cell cycle and apoptosis, cells were treated with Krishan’s reagent (0.05 mg/ml propidium iodide (PI), 0.1% Na citrate, 0.02 mg/ml ribonuclease A, 0.3% NP-40) for 30 min on ice and analyzed by flow cytometry, as described previously. [^4^](#_ENREF_4) Apoptosis was also analyzed by flow cytometric analysis of cells stained with Annexin V-PE and 7-AAD using PE annexin V Apoptosis Detection Kit I from BD Pharmingen (San Diego, CA, USA).

**Immunoprecipitation and Immunoblot Analyses**

For immunoprecipitation experiments, cells were lysed in a lysis buffer containing 1% Triton X-100, 20 mM Tris-HCl (pH 7.5), 150 mM NaCl, 1 mM EDTA, 1 mM sodium orthovanadate, 1 mM phenylmethylsulfonyl fluoride and 10 µg/ml each of aprotinin and leupeptin. Cell lysates were subjected to immunoprecipitation and immunoblotting as described previously.[^5^](#_ENREF_5) For immunoblot analysis of total cell lysates, samples were prepared by mixing an aliquot of cell lysates with an equal volume of 2X Laemmli’s sample buffer and heating at 100 °C for 5 min. The results shown are representative of experiments repeated at least three times.

**SUPPLEMENTARY REFERENCES**

1. Tohda S, Sato T, Kogoshi H, Fu L, Sakano S, Nara N. Establishment of a novel B-cell lymphoma cell line with suppressed growth by gamma-secretase inhibitors. *Leuk Res* 2006; **30:** 1385-1390.

2. Ngo VN, Young RM, Schmitz R, Jhavar S, Xiao W, Lim KH*, et al.* Oncogenically active MYD88 mutations in human lymphoma. *Nature* 2011; **470:** 115-119.

3. Oshikawa G, Nagao T, Wu N, Kurosu T, Miura O. c-Cbl and Cbl-b ligases mediate 17-allylaminodemethoxygeldanamycin-induced degradation of autophosphorylated Flt3 kinase with internal tandem duplication through the ubiquitin proteasome pathway. *J Biol Chem* 2011; **286:** 30263-30273.

4. Kurosu T, Ohki M, Wu N, Kagechika H, Miura O. Sorafenib induces apoptosis specifically in cells expressing BCR/ABL by inhibiting its kinase activity to activate the intrinsic mitochondrial pathway. *Cancer Res* 2009; **69:** 3927-3936.

5. Miura O, Nakamura N, Ihle JN, Aoki N. Erythropoietin-dependent association of phosphatidylinositol 3-kinase with tyrosine-phosphorylated erythropoietin receptor. *J Biol Chem* 1994; **269:** 614-620.

**Figure S1. Analyses of *MYD88* L265P mutation in patients with Waldenström macroglobulinemia by the allele specific PCR method.** Genomic DNA from bone marrow mononuclear cells from 5 patients with WM (1-5), including the patient with the novel MYD88 L265RPP mutation (1), as well as from BJAB (N) and TMD8 (P) cells were analyzed by the allele specific PCR method described in Materials and Methods. Positions of PCR products representing wild-type MYD88 and MYD88 L265P are shown.
